# Evidence of pediatric sepsis caused by a drug resistant *Lactococcus garvieae* contaminated platelet concentrate

**DOI:** 10.1080/22221751.2022.2071174

**Published:** 2022-05-23

**Authors:** Luna Colagrossi, Valentino Costabile, Rossana Scutari, Marilena Agosta, Manuela Onori, Livia Mancinelli, Barbara Lucignano, Andrea Onetti Muda, Giada Del Baldo, Angela Mastronuzzi, Franco Locatelli, Guglielmo Trua, Mauro Montanari, Claudia Alteri, Paola Bernaschi, Carlo Federico Perno

**Affiliations:** a Department of Laboratories, Unit of Diagnostic Microbiology and Immunology and Multimodal Medicine Area, Bambino Gesù Children’s Hospital IRCCS, Rome, Italy; b Department of Oncology and Hemato-oncology, University of Milan, Milan, Italy; c Department of Pediatric Hematology/Oncology and Cellular and Gene Therapy, Bambino Gesù Children’s Hospital IRCCS, Rome, Italy; d Department of Transfusion Medicine, Bambino Gesù Children’s Hospital IRCCS, Rome, Italy

**Keywords:** Sepsis, pediatric sepsis, onco-hematology, blood transfusion, *Lactococcus garvieae*, pathogen transmission, metagenomic, drug resistance

## Abstract

Owing to an increasing number of infections in adults, *Lactococcus (L.) garvieae* has gained recognition as an emerging human pathogen, causing bacteraemia and septicaemia. In September 2020, four paediatric onco-hematologic patients received a platelet concentrate from the same adult donor at Bambino Gesù Children’s Hospital IRCCS, Rome. Three of four patients experienced *L. garvieae* sepsis one day after transfusion. The *L. garvieae* pediatric isolates and the donor’s platelet concentrates were retrospectively collected for whole-genome sequencing and shot-gun metagenomics, respectively (Illumina HiSeq). By de novo assembly of the *L. garvieae* genomes, we found that all three pediatric isolates shared a 99.9% identity and were characterized by 440 common SNPs. Plasmid pUC11C (conferring virulence properties) and the temperate prophage Plg-Tb25 were detected in all three strains. Core SNP genome-based maximum likelihood and Bayesian trees confirmed their phylogenetic common origin and revealed their relationship with *L. garvieae* strains affecting cows and humans (bootstrap values >100 and posterior probabilities = 1.00). Bacterial reads obtained by the donor’s platelet concentrate have been profiled with MetaPhlAn2 (v.2.7.5); among these, 29.9% belonged to Firmicutes, and 5.16% to Streptococcaceae (>97% identity with *L. garvieae*), confirming the presence of *L. garvieae* in the platelet concentrate transfusion. These data showed three episodes of sepsis for the first time due to a transfusion-associated transmission of *L. garvieae* in three pediatric hospitalized hematology patients. This highlights the importance to implement the screening of platelet components with new human-defined pathogens for ensuring the safety of blood supply, and more broadly, for the surveillance of emerging pathogens.

## Introduction

*Lactococci* are Gram-positive, catalase-negative, facultative anaerobic cocci in short chains or pairs traditionally considered to be of low virulence to human beings [1]. Among them, *Lactococcus (L.) garvieae* species was first described in the 1950s in Japan, when it was discovered in a rainbow trout farm [2,3]*.* It can be found in a vast variety of environments due to its ability to adapt easily. It has been isolated from aquaculture, rivers, and sewage waters. The host range of *L. garvieae* is not limited to aquatic species, but is also associated with subclinical mastitis in cows and water buffalos, and pneumonia in pigs [4,5]. Previous studies have reported an association between *L. garvieae* infection and contaminated food, such as raw milk, cheese, vegetables, cereals, and meat [6,7].

The first case of *L. garvieae* human infection was observed in 1991 [8]. Since then, the relevance and clinical significance in humans have increased. After the first documented case, more than 30 new cases of *L. garvieae* infection have been described in literature [6,9–11]. Due to the increasing number of human clinical infections*, L. garvieae* has gained recognition as an emerging human pathogen. Among the reported cases, majority have been associated with bacteraemia with infective endocarditis among elderly and immunocompromised patients [9]. Other clinical syndromes include spontaneous septicaemia, liver abscess, bone infections, diverticulitis, and secondary peritonitis [3,9,11]. It has also been observed to mainly cause urinary tract infection (UTI) [1]. However, the true incidence of the disease is difficult to assess because of the morphological and biochemical similarities to other Gram-positive cocci like *Enterococcus spp*. and *Streptococci* [9,12].

In 2018, the first case of transfusion-transmitted *L. garvieae* resulting from a platelet transfusion in an adult hospitalized individual was described [13]. Here, we describe a cluster of transmission, with three cases of sepsis in onco-hematologic pediatric patients, derived by transfusion-transmitted *L. garvieae* from platelet concentrates (PCs) of the same donor.

## Material and methods

### Study population

On 31 August 2020, blood donation was collected from a healthy 59-year-old Italian female at IRCCS Bambino Gesù Children’s Hospital, in Rome, Italy. PCs were obtained by apheresis collection. The PCs were confirmed to have no abnormalities by visual inspection at the blood centre and again at the bedside just before starting the transfusion. These PCs were transfused to four paediatric patients at different timetable: patient 1 was transfused one day after blood donor’s collection, while patients 2–4 were transfused on day 2 after blood collection (hour:minute: 14:04, 14:32, and 16:36, respectively).

### Data and samples collection

Age, sex, data regarding antibiotic therapies, comorbidities, and clinical conditions (body temperature, laboratory tests, and vital parameters) on the day of transfusion were collected for each patient.

At least one blood culture (one bottle for aerobic growth and one for anaerobic growth) was drawn for each patient after the transfusion as part of routine care. The Microbiology Unit of the Hospital processed the samples. According to the standard laboratory operating procedures, the identification of microorganisms was performed by MALDI-TOF mass spectrometry. As no breakpoints for antibiotic susceptibility have been determined for *Lactococcus spp*., antibiotic susceptibility was analyzed by the Vitek system according to the EUCAST guidelines for *Streptococci*. Antibiotic susceptibility was analyzed against the glycopeptides, teicoplanin and vancomycin; the penicillins, ampicillin and penicillin G; the cephalosporin, ceftriaxone; the lincosamide, clindamycin; the macrolide, erythromycin; the aminoglycoside, gentamycin; and the fluoroquinolone, levofloxacin.

Sepsis disease is defined as a suspected or proven infection with at least two of the following criteria: abnormal temperature (>38.5°C or <36°C), abnormal white-blood-cell count (elevated [>20,000 × 10⁹ per L] or decreased [<4000 × 10⁹ per L] for age), tachycardia or bradycardia, or tachypnoea [14], C-reactive protein greater than 15 mg/L, and serum procalcitonin increase above 0.05 ng/mL [15].

### Ethics approval

This study was conducted with respect to the Helsinki Declaration, and all the participants (parents) signed an informed consent to allow the use of clinical data for research purposes. Ethical approval was obtained from the Ethics Committee of Bambino Gesù Children’s Hospital in Rome, Italy (reference ID 2602/2021).

### Whole-genome sequencing and metagenomic analysis of donor’s platelet concentrates

#### Extraction and sequencing

Microbial DNA was extracted from the positive cultures and the donor’s blood samples using the QIAamp DNA Microbiome Kit (Qiagen, Germany) according to the manufacturer’s instructions. DNA was quantified using the Qubit fluorometer. Libraries were obtained by QIAseq FX Single Cell DNA Library kit and DNAs were paired-end sequenced (2 × 150 bp) using Illumina HiSeq (Illumina, USA). All bacterial sequence data obtained were screened for the evidence of baseline quality control for short and low sequences. The quality check was done with FastQC v0.72.14. Adaptors were clipped and quality trimmed using FASTP 0.20.1 using default parameters (Q > 30). Quality trimmed paired reads were assembled into contigs using ABYSS v2.0, a de-novo assembler based on de Bruijn graph path reconstruction [16]. K-mer size optimization has been reached by comparing QUAST indices [17]obtained by assembling bacterial genomes with different k-mer sizes and by selecting the one returning the best score.

#### Phylogenetic analysis

To explore a possible clonal origin of the three *L. garvieae* strains isolated from paediatric patients, the *L. garvieae* genomes were compared to 20 publicly available *L. garvieae* sequences by both maximum likelihood and Bayesian approaches. Four publicly available additional *Lactococcus (L.) lactis* sequences were used as an outgroup. The characteristics of the 24 reference genomes are described in Table S1. PhaME software was used to perform the core single nucleotide polymorphism (SNP) genome typing [18]. The core SNP alignment was indeed composed of 27 sequences 62,990 nucleotide long. Phylogenetic relatedness was first analyzed by the maximum likelihood (ML) method using iqTree2 [19], under the nucleotide substitution GTR+I+G4 model [20] and 1000 bootstrap replicates. The results were confirmed by a Bayesian inference analysis through BEAST v.1.10.4 [21] by setting a chain length of 100 million of states under a strict molecular clock model and the GTR+I+G4 substitution model. The core SNP multiple alignment composed of the three *L. garvieae* strains isolated from paediatric patients plus 24 publicly available *L. garvieae* and *L. lactis* strains used for phylogenetic analysis is available at d oi: 10.5281/zenodo.6473676. To better appreciate the conserved residues between Enterococcus, Streptococcus, and Lactococcus species, the core SNP multiple alignments composed of (i) the three *L. garvieae* strains isolated from paediatric patients plus 24 publicly available *L. garvieae*, *L. lactis*, and *Streptococcus (S.) pneumoniae* strains, (ii) the three *L. garvieae* strains isolated from paediatric patients plus three publicly available *L. lactis*, *S. canis*, and *Enterococcus (E.) raffinosus* strains are available at the same d oi: 10.5281/zenodo.6473676.

#### Functional analysis

Resistance genes, plasmids, virulence factors, insertion sequence elements (IS), and phages were annotated using BLASTN e BLASTX against several specific databases, including CARD, ARDB, PlasmidFinder, ResFinder, VirulenceFinder, ICEberg, VFDB, ISFinder, phySPY, PHASTER, and PLSDB [7,22–26]. Outputs have been parsed to discard all the hits with a BIT score <300, coverage <65%, and similarity <70%. The remaining hits have been ordered by position, and in case of overlapping matches, the ones with the highest BIT score have been selected.

#### Metagenomics

Sequencing data pre-processing of the donor’s blood sample was performed by FASTP 0.20.1. Human reads were mapped against the human reference genome hg19 using bowtie2 and removed by *samtools* tools [27]. The host-filtered microbial reads were taxonomically profiled using MetaPhlAn2 (version 2.7.5) [28,29].

## Results

### Patients’ characteristics

In September 2020, four paediatric onco-hematologic patients received PCs from the same irradiated sample, prepared by a single-donor (a healthy 59-year-old Italian female) plateletpheresis at Bambino Gesù Children’s Hospital (OPBG), Rome, Italy.

Three patients were males, with a median (interquartile range, IQR) age of 8 (7–9) years (Table 1). Three of them were affected by acute lymphocytic leukemia (ALL) (patients 1–3), while one was affected by metastatic neuroblastoma (patient 4). All were immunocompromised (Table 1).

**Table 1.** Pediatric patients’ characteristics.

	Patient 1	Patient 2	Patient 3	Patient 4
Sex	Female	Male	Male	Male
Age, years	8	9	4	9
Nationality	Italian	Italian	Italian	Italian
Disease	ALL	ALL	ALL	Metastatic neuroblastoma
PCs transfusion (day/month/year; hour:minute)	01/09/2020; 16:53	02/09/2020; 14:04	02/09/2020; 14:32	02/09/2020; 16:36
*At PCs transfusion*:				
Immunosuppression	Yes	Yes	Yes	Yes
White blood cells, cells/µL	100.0	800.0	7600.0	700.0
Red blood cells, 10^6^cells/µL	2.5	2.3	2.8	3.5
Hb, g/dL	8.0	7.3	8.3	9.6
Ht, %	24.3	21.8	23.3	27.3
MCV, fL	96.5	96.0	84.1	77.1
MCH, pg	31.7	32.0	30.0	27.2
MCHC, g/dL	32.9	33.4	35.6	35.3
RDW, %	18.3	22.2	13.3	13.1
HDW, g/dL	3.4	4.1	3.3	3.1
Platelets, number/µL	20000.0	135000.0	38000.0	29000.0
Neutrophils, %	31.8	36.6	92.2	62.3
Lymphocytes, %	61.2	33.2	2.9	24.8
Monocytes, %	1.2	12.3	3.7	5.9
Eosinophils, %	2.4	1.0	0.3	4.0
Basophils, %	0.0	0.6	0.0	0.3
Leukocytes, %	3.5	11.7	0.8	2.7
*Lactococcus garvieae* isolation from blood culture (day/month/year; hour:minute)	–	03/09/2020; 20:29	03/09/2020; 11:14	03/09/2020; 09:28
*Symptoms after positive blood culture:*				
Fever, celsius	39.2	39.7	40.0	38.2
Chills	Yes	Yes	Yes	Yes
Heart rates, bpm	110	140	160	150.00
Respiratory rate, breaths/min	22	20	30	22
Arterial blood pressure, mmHg	99/64	117/74	80/40	110/70
Procalcitonin, ng/Ml	18.5	121.6	61.9	356.0
CRP, mg/dL	2.8	15.3	5.9	11.8
Empiric antimicrobial therapy	INN-Tigecycline; Amikacin; β-lactams	INN-Tigecycline; Amikacin; INN-ceftazidime/avibactam	INN-Tigecycline; Amikacin; β-lactams; Quinolones; Metronidazole;	INN-Tigecycline; Amikacin*; Quinolones

ALL: Acute lymphocytic leukemia; PCs: Platelet concentrates; Hb: Hemoglobin; Ht: Hematocrit; MCV: Mean Corpuscular Volume; MCH: Mean Corpuscular Hemoglobin; MCHC: Mean Corpuscular Hemoglobin Concentration; RDW: Red Cell Distribution Width; HDW: Hemoglobin Distribution Width; CRP: C-reactive Protein; INN: International Non-proprietary Name. *Replaced by Gentamycin one day later.

**Figure 1.**
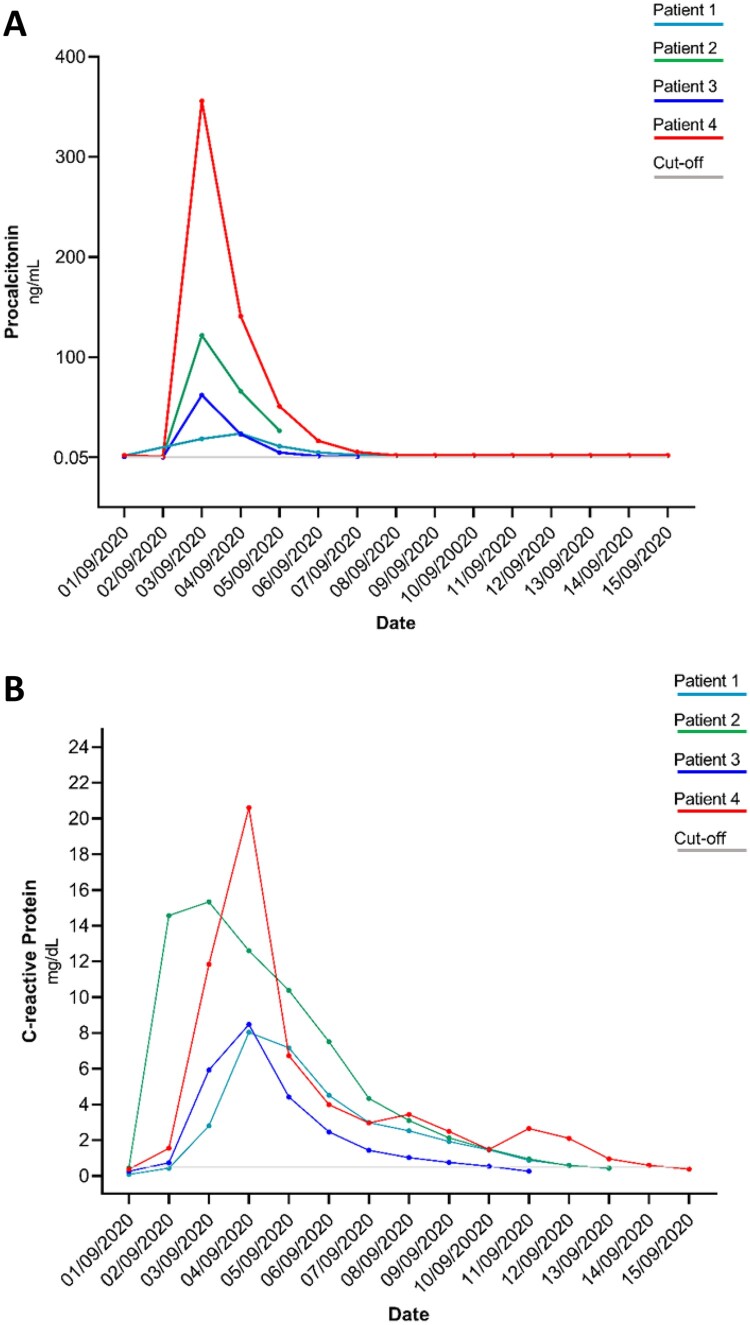
Procalcitonin (Panel A) and C-reactive protein (Panel B) kinetics in the four paediatric patients receiving the platelet concentrates. All patients received platelet concentrates on 2 September 2020 except patient 1 who received the concentrates on 1 September 2020. Isolation of *L. garvieae* occurred for all patients (patients 1, 2, and 3) on 3 September 2020. Cut-off: normal value. Procalcitonin normal value <0.05 ng/mL; C-reactive protein normal value <0.50 mg/dL.

Approximately 24 h after transfusion, body temperature, C-reactive protein, and procalcitonin levels have been altered in all four patients, reaching elevated values (>38°C, >5 mg/dL, and >60 ng/mL, respectively) in three out of four patients (patients 2–4). Accordingly, a clinically defined sepsis was diagnosed in patients 2, 3, and 4 (Table 1 and Figure 1), while patient 1 experienced an acute self-limited illness. An empiric antimicrobial therapy mainly composed of tigecycline and aminoglycosides (Table 1) was administered to each patient as soon as clinical manifestations were evident. In all patients, the vital signs returned to physiological ranges in a median (IQR) time of 4.5 (3.5–5.75) days.

### Phylogenetic relatedness of *L. garvieae* isolates

*L. garvieae* was isolated from the blood culture of all three patients developing sepsis (patients 2–4) 24 h post PCs transfusion, confirming the bloodstream infection. The blood culture of patient 1 resulted negative, confirming the self-limited illness.

The antimicrobial susceptibility testing defined resistance to the lincosamide clindamycin and penicillin G for all isolates (minimal inhibitor concentration [MIC] values: >256 and ≥0.5, respectively).

WGS experiment returned a total of 4.15 GB of Illumina 2 × 150 bp data by the sequencing of three *L. garvieae* isolates. De novo genome reconstruction retrieved an average genome size of 2.06 million base pairs with a GC content of 37.9%. The three *L. garvieae* sequences are available in the European Nucleotide Archive (ENA) under the following BioSample accession numbers: SAMEA12289652, SAMEA12289653, and SAMEA12289654.

Looking at the genome content, the three strains were characterized by 440 common SNPs, 44 of them were never described before (Table S2). Of these 43 SNPs, 19 were non-synonymous, and thus, involved in amino acid modifications in different *L. garvieae* enzymes.

**Figure 2.**
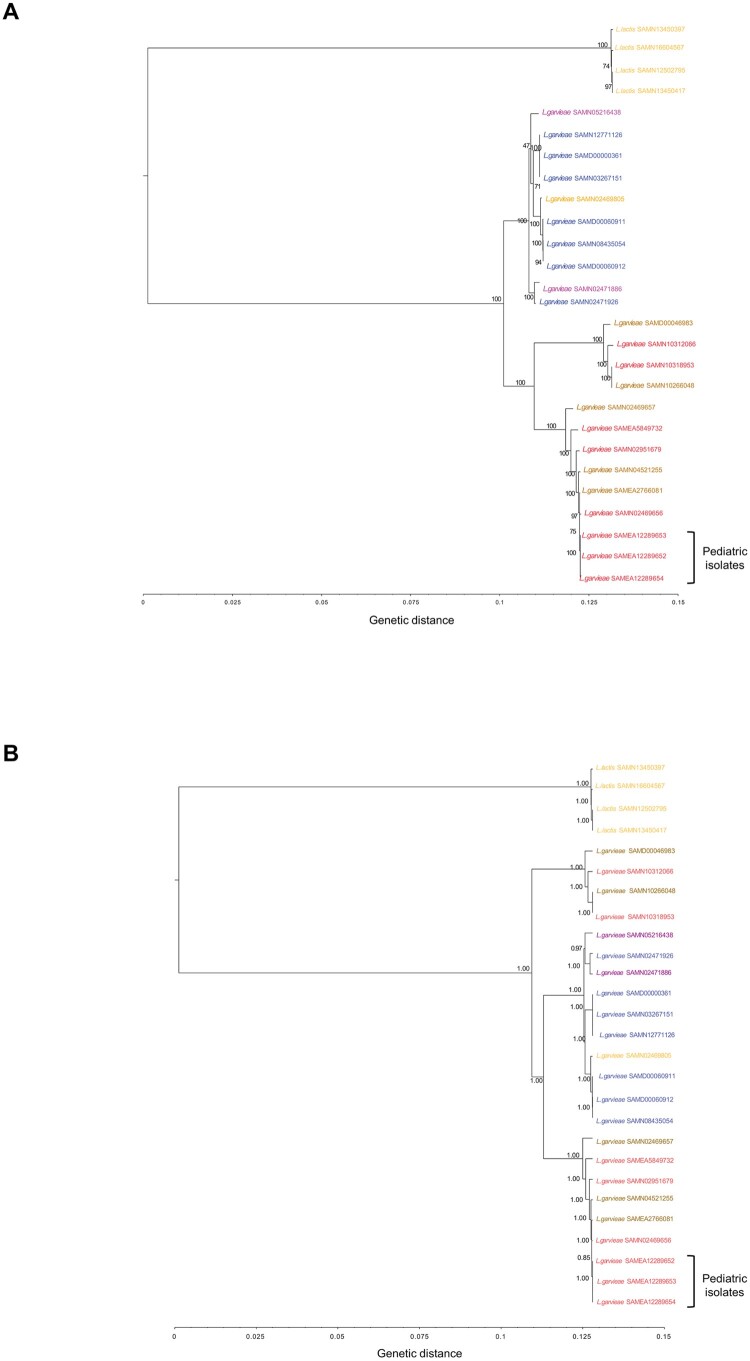
Estimated maximum likelihood (A) and Bayesian (B) phylogenies based on coreSNP genome of the three *L. garvieae* strains isolated from pediatric patients at Bambino Gesù Pediatric Hospital, IRCCS. Representative 20 *L. garvieae* and 4 *L. lactis* strains retrieved by public databases were also included to obtain an alignment of 27 coreSNP genomes 62,990 nucleotides long. The *L. lactis* and *L. garvieae* strains were annotated according to collection source (Orange: Dairy products, Blue: Fishes, Brown: Cow, Red: Humans; Other: Purple). BioSample accession numbers defined the strains. Panel (A) reported the ML tree, inferred using iqTree2 under the nucleotide substitution GTR+I+G4 model and 1000 bootstrap replicates. Panel (B) reported the Bayesian tree, performed by BEAST v.1.10.4 setting a chain length of 100 million states under a strict molecular clock model and the GTR+I+G4 substitution model. Bootstrap values and posterior probabilities are reported along the branches of the trees.

The ML tree (based on the core SNP genomes of the three pediatric *L. garvieae* isolates plus 24 publicly available *L. garvieae* and *L. lactis* sequences, Table S1) showed that the three isolates shared a 99.9% identity, and clustered together within a bootstrap of 100% in the subtree of human and cow *L. garvieae* strains (Figure 2(A)). The common origin of the three *L. garvieae* strains and their strong relatedness with human and cow strains were further confirmed by the Bayesian inference as suggested by the topology of the tree and the clusters’ posterior probabilities equal to 1.00 (Figure 2(B)).

Of note, the isolates described in this work exhibit a genetic relatedness with *L. garvieae* isolates from meat and dairy products made with raw milk, previously pointed as the potential sources of human *L. garvieae* infection [5].

### Virulence factors and resistance genes characterizing the *L. garvieae* isolates

The isolates hold several virulence factors, such as hemolysin III family proteins, adhesin gene clusters, siderophores, and sortases (Table 2), suggesting a role of these strains in contributing to the pathogenic manifestations and the reported illnesses of the three pediatric patients.

**Table 2.** Putative virulence factors and resistance genes identified in the three *L. garvieae* strains isolated from pediatric patients at Bambino Gesù Pediatric Hospital, IRCCS.

Gene product	Accession number and locus	Identity (%)	Length (nt)	E-value*	BitScore**
*Chromosomal content*					
Hemolysin III related protein	NC_017490 region:331642-352295	98.61	217	<1E-100	424
U32 family peptidase [Collagenase]	NC_017490 region: 1699976-1701265	99.53	429	<1E-100	887
MucBP domain-containing protein LCGL 1005	AFCC01000046 region:18820-20355	97.65	680	<1E-100	1321
WxL surface protein	AFCD01000027 region:17404-18222	89.35	629	<1E-100	1019
LPxTG surface protein (Cna B-domain) (orf 25)	HM852551 WP_019291416.1	99.81	2,115	<1E-100	4031
Sortase A	NC_017490 region: 13005315-1306046	96.29	243	<1E-100	443
Adhesins gene cluster - WxL surface protein	NC_017490 region: 878477-872476	96.55	203	<1E-100	389
Adhesins gene cluster - Cell surface putative DUF916 and DUF3324 domain containing protein	DUF916 and DUF3324	93.17	337	<1E-100	629
Adhesins gene cluster - Putative surface protein (COG4713 domain)	HM852557 AFCC01000021 region: 19917-21412	98.95	190	<1E-100	382
Pili specific sortase SpaH/EbpB family LPxTG-anchored major pilin	WP 100222480.1 in AFCD01000058 region: 28828-35485	98.61	502	<1E-100	914
ABC transporter ATP-binding protein LCGL 1621	NC_017490 region: 1594232-1596948	92.64	258	<1E-100	460
ABC transporter substrate-binding protein LCGL 1622		92.62	339	<1E-100	565
ABC transporter substrate-binding protein LCGL 1623		92.01	313	<1E-100	588
Iron ABC transporter permease LCGL 0527	NC_017490 region: 544393-548003	98.85	260	<1E-100	495
Iron ABC transporter permease LCGL 0528		93.98	316	<1E-100	429
Iron ABC transporter permease LCGL 0529		94.90	294	<1E-100	516
Iron-hydroxamate ABC transporter LCGL 0530		98.40	313	<1E-100	605
Ferrous iron transport protein B LCGL RS10540	NC_017490 region: 106587-109166	97.11	693	<1E-100	1321
Phospho-sugar mutase (Phosphoglucomutase) LCGL 1596	NC_017490 region: 1558243-1559955	98.95	570	<1E-100	1158
Enolase [phosphopyruvate hydratase]	NC_017490 region: 1489407-149070	100.00	429	<1E-100	867
Glyceraldehyde-3-phosphate dehydrogenase LCGL RS19790	NC_017490 region: 1928003-1929013	100.00	336	<1E-100	671
Superoxide dismutase LCGL 0285	NC_017490 region: 289210-289818	99.01	202	<1E-100	385
FAD-dependent oxidoreductase LCGL 0664	NC_017490 region: 678241-679578	99.10	445	<1E-100	907
Glycoside hydrolase family 1 protein	NZ_CP065637 region: 716031-717467	99.8	478	<1E-100	487
*Antibiotic resistance genes*					
Lsa(D)^a^	MH473150.1	90.76	1493	<1E-100	1993
mdt(A)^a^	WP_176490218.1	88.87	1258	<1E-100	1546
vanZ-like domain	NZ_CP065637 region: 571963-572424	93.49	1045	<1E-100	1554
penicillin-binding protein 1A^b^	GFO52038.1	97.12	694	<1E-100	1316
penicillin-binding protein 2A^b^	GFO51196.1	98.29	762	<1E-100	1453
putative penicillin-binding protein PbpX^b^	CEF50305.1	99.68	314	<1E-100	619
penicillin-binding protein 2B^b^	GFO51136.1	97.35	718	<1E-100	1329
penicillin-binding protein 1B^b^	GFO51088.1	97.65	807	<1E-100	1483
*Plasmid content (pUC11C)*					
Sortase C (n2)	ARE12311	99.58	1179	<1E-100	2150
IS6 family transposase	ARE12304	100	96	6.69E-46	178
mobilization/filimentation protein Fic	ARE12315	98.34	603	<1E-100	1059
Resolvase: Exogenous DNA integration	ARE12303	90.99	555	<1E-100	749
SpaH/EbpB family LPXTG-anchored major pilin	WP_082225092.1	98.6	501	<1E-100	911
VWA domain c protein	WP_082225091.1	98.29	1341	<1E-100	2289
*Insertion sequences*					
IS6 family – IS1216E^c^	IS1216E	98.0	132	<1E-100	238
IS6 family – ISS1N^c^	ISS1N	98.0	46	<1E-100	61.9

Putative virulence factors were annotated using BLASTN e BLASTX. Identity was estimated against the reference genome for each gene product. Only hits with BIT score >300, coverage >65%, and identity >80% were considered. ^a^Lsa(D) is a specific resistance factor providing tolerance to lincosamides, while mdt(A) is a drug antiporter responsible for aspecific resistance to tetracyclines and macolides; both of them belong to chromosomal genetic content. ^b^Penicillin binding protein (PBP) are chromosomal genes that are normally involved in cell wall biosynthesis; their full resistance mechanism is achieved by the actions of auxiliary genes mainly found in Staphylococcus pathogenic strains (holding chromosomal *mec* cassette); they provide only partial resistance in other bacterial genera. ^c^Insertion Sequences 6 family have been characterized as important factors determining bacterial genome shaping. In fact, they are responsible for exogenous genetic content integration via IS6 family transposase action (found to be part of pUC11C plasmid). *E-value index returns the probability to get a match by chance. **BIT score is the match reliability index.

Antimicrobial resistance profile is promoted in the three isolates by chromosomal lsa(D) gene, recently detected in *L. garvieae* fish pathogenic strain and described as a novel factor for resistance to lincosamide, pleuromutilins, and streptogramins [30]. It acts as an ATP-dependent active transport and does not require additional cofactors to confer full resistance to upper mentioned antibiotics. The multidrug transporter Mdt(A) gene, originally described as a plasmid-dependent antimicrobial resistance factor in *E. coli* and *L. lactis* [31], and recently found in the genomic content of *L. garvieae* [32], was also shared in all the three isolates. Different from *E. coli* and *L. lactis*, this enzyme does not confer decreased susceptibility to erythromycin or tetracycline in *L. garvieae* species, probably due to two amino acid mutations in the C-motifs of Mdt(A), known to suppress the resistance phenotype of Tet(K) and Tet (L) [33,34].

The intermediate penicillin resistance found by the antimicrobial susceptibility test was probably promoted to a group of chromosomal penicillin binding proteins (PBP1a, PBP1b, PBPx, PBP2a, PBP2b, Table 2) normally involved in wall cell biosynthesis [35], and thus, capable of reacting with β-lactams. The intermediate, instead of being fully resistant to penicillin, can be explained by the absence of an auxiliary set of protein and regulatory elements encoded by a cassette chromosome called *mec* found in most of the penicillin-resistant *Staphylococci*, but absent in other non-pathogenic species [36].

Of note, the genome content analysis also revealed a vanZ-like domain, known to be present in the genomes of clinically relevant bacteria, such as *Bacillus*, *Streptococcus*, *Enterococcus*, and *Clostridium*, and decreases their sensitivity to some lipoglycopeptide antibiotics, but not vancomycin [37,38].

Non-chromosomal genomic content in our isolates is represented by the plasmid pUC11C (Table 2) [39], known to encode two class C sortases, which are commonly involved in pilus biosynthesis [40,41].

The three *L. garvieae* genomes also contained the PLg-TB25 temperate prophage (coverage: 71.19%), recently described as a non-virulent prophage from a dairy strain of *L. garvieae* [42], thus confirming the genetic relatedness of the three *L. garvieae* strains highlighted by the phylogenetic tree. This prophage carries with it several enzymes, such as resolvase and helicase, promoting exogenous DNA integration into the bacterial chromosome, and thus, paying an active role in acquiring and fixing genetic elements going under positive selection.

### Donor’s platelet concentrates characterization

To confirm the source of *L. garvieae* infection, the microbial reads obtained by the donor’s PCs were profiled – thanks to MetaPhlAn2 (v.2.7.5). After removing low-quality and host genome reads, we obtained 5,459,177 reads with a quality score >30. The 4.98% (number of reads: 271,810) were properly classified as bacteria (*n* = 64,145), viruses (*n* = 200,356), or eukarya (*n* = 7,309), while 95.2% remained unclassified. Among the bacterial reads, 29.9% (number of reads: 19,154) belonged to Firmicutes, and 5.16% (number of reads: 3310) defined Streptococcaceae. The remaining bacterial reads mainly belonged to Proteobacteria (number of reads: 38,127).

All Streptococcaceae reads (ENA accession number: ERS9886497) shared a >97% homology with *L. garvieae* isolates, thus confirming the presence of *L. garvieae* in the PCs transfusion.

## Discussion

Here, we describe three cases of sepsis-related to the drug-resistant *L. garvieae* transmission in three onco-hematologic pediatric patients caused by a platelet transfusion obtained by the same healthy adult donor. The fourth patient, who had received a transfusion first, developed a self-limited illness, accompanied by a blood culture that remained negative. To our knowledge, this is the first report that described a clinically defined sepsis in a pediatric setting caused by this emerging human pathogen during a blood transfusion procedure.

The cases of *L. garvieae* infection in humans described so far are characterized by a favourable clinical course and regard manifestations such as endocarditis, septicaemia, urinary tract infection, peritonitis, and liver abscess [3,9,11]. *L. garvieae* was also the cause of bacterial contamination of PCs [43,44], and thus, represents a serious problem in transfusions, as demonstrated by the first case of sepsis caused by this transfusion-transmitted pathogen [43].

In this regard, the safety of the blood supply, including bacterial contamination of platelet products and transfusion-transmission risks associated with emerging pathogens [45,46] continues to represent a challenge for clinical blood centers. In the pediatric setting, sepsis without source account for 3.4%–13.6% of cases seen in emergency departments [47], and all are characterized by a challenging diagnosis. Today, there are certain technologies that mitigate this risk either as commercially available products or as investigational protocols. These include pathogen-reduction technology (PRT) for apheresis platelets and plasma, rapid tests for bacterial detection in PCs, and investigational screening assays for emerging pathogens. The implementation of these technologies has enhanced the safety of the blood supply in the last years [48], even if full prevention of transfusion-related bacterial infection cannot be completely achieved. In 2019, according to the latest FDA report, approximately 1.9 million apheresis platelets were transfused, and one death due to bacterial contamination occurred [49].

Here, the three cases of sepsis in pediatric recipients developed 24 h after PCs transfusion. All three patients (patients 2–4) were characterized by a peak fever and a significant C-reactive protein and procalcitonin increase (Figure 1). Consistent with clinical manifestations, the blood culture revealed *L. garvieae* infection in all three patients. The genome content analysis of *L. garvieae* isolates from the three blood cultures suggested their clonal origin and a well-defined homology with *L. garvieae* derived from meat and dairy products [7]. The source and the transmission chain were revealed by the metagenomics analysis that confirmed the presence of *L. garvieae* traces in PCs.

Even if all patients had a favourable clinical course after a fully active antimicrobial therapy composed mostly of tigecycline and aminoglycosides (Amikacin), the illnesses reported in three out four patients after transfusion were typical of a pathogenic microorganism invasion.

In view of this and other reports that described sepsis associated with *L. garvieae* infection [11,13,50,51], the knowledge of virulence factors and resistance mechanisms associated with the *Lactococcus* genera and *L. garvieae* species is detrimental.

Here, we implemented the knowledge of the virulent *L. garvieae* circulating strains by providing a capillary description of chromosomal and extrachromosomal content of these bacteria, focusing the attention to all factors that might confer a selective advantage in host invasion [7]. Indeed, the three strains hold the virulence factors necessary to survive and feed in iron rich-environment, like human blood [52]. Collected strains also shared several groups of adhesins, haemolysin, fibronectin-binding proteins, and penicillin acylase that actively promote bacterial colonization of mucosal tissues [53,54], and thus, increase the chance of bacteria being present in blood transfusable components [52,54]. Some adhesins such as MucBP domain-containing protein LCGL 1005 also confer to the bacteria the ability to form biofilm and to escape immune surveillance systems [55]. Furthermore, the presence of WxL domain-containing proteins (already characterized in *Enterococcus faecium*) increases the *L. garvieae* ability to overcome the osmotic stress and to aggregate in a complex population [56].

We also identified chromosomal contents conferring drug resistance to lincosamides (IsaD gene) and penicillins (penicillin binding proteins) (Table 2), and to some lipoglycopeptide antibiotics [31,35,37,38,57], thus providing the genetic basis of the antimicrobial susceptibility testing results, that defined resistance to clindamycin and intermediate resistance to penicillin G. These results also confirmed the drug-resistant genetic backbone of the *L. garvieae* isolated in the three pediatric patients.

Even if the three cases described here support the circulation of drug-resistant *L. garvieae* strains in humans and its potential role as a human pathogen, more insights and evidence are needed to better define *L. garvieae* pathogenicity and related outcomes, and thus, to guide its significance in clinical practice. Most efforts in WGS are also needed to better characterize *L. garvieae* circulation in pediatric and adult settings.

Regarding transfusion-transmitted infection risk, although four patients had received the same platelet concentrate bag, only three developed sepsis. A discriminating factor that can justify the different grades of illness is the time of transfusion administration.

According to our hospital procedure [58], PCs are generally stored on a platelet shaker at room temperature (22 ± 2°C) before being transfused. Due to the ability of *L. garvieae* to grow at temperatures as low as 10°C in red blood cells [59], the platelets storage at room temperature could have provided suitable conditions for bacterial proliferation [48].

In line with this evidence, we observed that the first patient who received the transfusion was the only one who did not develop sepsis. On the contrary, the patient who last received the platelet concentrate bag was the first to have a positive blood culture. Interestingly, this patient was also characterized by a higher level of procalcitonin than the others (Figure 1), providing further evidence that in the case of bacterial contamination, the longer is the time of platelets storage, the higher is the risk to transmit high bacterial load [60]. It is worth knowing that the irradiation performed according to internal hospital procedures is used to prevent the Graft *versus* Host Disease (GvHD), but is not able to prevent bacterial growth as stated in the National Guidelines [58].

In conclusion, despite advances in donor screening and infectious disease testing, the risk of transfusion-transmitted infections continues to be of particular concern, as defined by the three episodes of sepsis due to a transfusion-associated transmission of drug-resistant *L. garvieae* in pediatric hospitalized onco-hematologic patients. This highlights the importance to implement the screening of platelet components with new human-defined pathogens to ensure the safety of blood supply, and more broadly, for surveillance of emerging pathogens.

## Supplementary Material

Supplemental Material
