# Supplementary material for: Evidence of pediatric sepsis caused by a drug resistant Lactococcus garvieae contaminated platelet concentrate
Source: Emerg Microbes Infect. 2022 May 23;11(1):1325–34. doi: 10.1080/22221751.2022.2071174 (PMC9132404; doi:10.1080/22221751.2022.2071174)
Supplement: Supplemental Material [file TEMI_A_2071174_SM4915.zip › Supplemental file/8_revTableS1.docx]

**Table S1. Representative *Lactococcus lactis* and *Lactococcus garvieae* sequences used for Maximum Likelihood and Bayesian trees.**

|  | **Strain code** | **BioSample Accession Number** | **Host** | **Country** |
| --- | --- | --- | --- | --- |
| **Lactococcus garvieae** | ATCC49156 | SAMD00060911 | Fish | Japan |
|  | JJJN1 | SAMN08435054 | Fish | South Korea |
|  | Lg2 | SAMD00060912 | Fish | Japan |
|  | 21881 | SAMN02469656 | Human | Spain |
|  | DB24910 | SAMN10312066 | Human | Singapore |
|  | LGilsanpaikGS201105 | SAMN02951679 | Human | South Korea |
|  | MGYGHGUT00230 | SAMEA5849732 | Human | China |
|  | UNIUD074 | SAMN02471926 | Fish | Italy |
|  | M14 | SAMEA2766081 | Dairy products | Algeria |
|  | NBRC_100934 | SAMD00000361 | Cow | Japan |
|  | 122061 | SAMD00046983 | Fish | Japan |
|  | CCUG_32208T | SAMN12771126 | Cow | Sweden |
|  | CT2 | SAMN10318953 | Fish | Singapore |
|  | DM12426 | SAMN10266048 | Human | Singapore |
|  | DSM_20684 | SAMN03267151 | Cow | China |
|  | 8831 | SAMN02469657 | Fish | Spain |
|  | KS1546 | SAMN04521255 | Cow | Kosovo |
|  | M79 | SAMN05216438 | Cow | USA |
|  | Tac2 | SAMN02471886 | Turkey | Italy |
|  | TRF1 | SAMN02469805 | Rattlesnake | USA |
| **Lactococcus lactis** | SD96 | SAMN12502795 | Dairy products | Denmark |
|  | FDAARGOS_887 | SAMN13450417 | Dairy products | NA |
|  | DRC3 | SAMN16604567 | Dairy products | Australia |
|  | FDAARGOS_867 | SAMN13450397 | Dairy products | NA |

‘NA’: Not Available
