# Supplementary material for: Evidence of pediatric sepsis caused by a drug resistant Lactococcus garvieae contaminated platelet concentrate
Source: Emerg Microbes Infect. 2022 May 23;11(1):1325–34. doi: 10.1080/22221751.2022.2071174 (PMC9132404; doi:10.1080/22221751.2022.2071174)
Supplement: Supplemental Material [file TEMI_A_2071174_SM4915.zip › Supplemental file/9_revTableS2.docx]

**Table S2.** **The 43 Novel Single-Nucleotide Polymorphism (SNPs) shared by the three *L. garvieae* strains isolated from pediatric patients at Bambino Gesù Pediatric Hospital, IRCCS.**

| **SNP position** | **SNPs** | **Codon** | **Amino acid** | **Synonymous (S)/Non-Synonymous (NS)** | **Gene product** |
| --- | --- | --- | --- | --- | --- |
| 4288 | A>T | TTA>TAA | L>* | NS | hypothetical protein |
| 5045 | C>T | TAC>TAT | Y | S | penicillin-binding protein |
| 7738 | NAR |  |  |  |  |
| 10202 | NAR |  |  |  |  |
| 14985 | A>G | ACA>GCA | T>A | NS | metal ABC transporter substrate-binding protein |
| 15932 | C>T | GTT>ATT | V>I | NS | MFS transporter |
| 17900 | NAR |  |  |  |  |
| 17954 | T>C>A | CTA>CTG>CTT | L | S | SMC family ATPase |
| 18805 | A>G | GCT>GCC | A | S | 30S ribosome-binding factor RbfA |
| 21482 | A>C | AAG>CAG | K>Q | NS | zinc ribbon domain-containing protein |
| 23420 | A>C | ATA>CTA | I>L | NS | signal peptidase II |
| 27138 | C>T | AAC>AAT | N | S | fructose-bisphosphate aldolase |
| 29233 | G>A | ACC>ACT | T | S | MurR |
| 33091 | NAR |  |  |  |  |
| 33697 | T>C | CCT>CCC | P | S | folate family ECF transporter S component |
| 37215 | C>T | CGA>CAA | R>Q | NS | SMC family ATPase |
| 39500 | A>T>G>C | CCA>CCT>CCG>CCC | P | S | ribosome biogenesis GTPase YlqF |
| 41431 | NAR |  |  |  |  |
| 45109 | T>G | GGT>GGG | G | S | hydroxymethylglutaryl-CoA reductase |
| 55291 | T>C | AAA>AAG | K | S | PTS transporter subunit EIIC |
| 61820 | G>T | CCA>ACA | P>T | NS | polyribonucleotide nucleotidyltransferase |
| 62645 | G>A | AGT>AAT | S>N | NS | 23S rRNA |
| 66144 | G>A | GCA>GTA | A>V | NS | DUF177 domain-containing protein |
| 66915 | G>C | GGC>GGG | G | S | amino acid permease |
| 69819 | NAR |  |  |  |  |
| 74610 | A>C | GAT>GCT | D>A | NS | F0F1 ATP synthase subunit delta |
| 75819 | G>T | GCT>GAT | A>D | NS | ArgE |
| 79620 | NAR |  |  |  |  |
| 80298 | NAR |  |  |  |  |
| 83799 | NAR |  |  |  |  |
| 87477 | T>C>A | CAA>CAG>CAT | Q>H | NS | nicotinate phosphoribosyltransferase |
| 90973 | C>T | CGT>CAT | R>H | NS | phosphate acetyltransferase |
| 90978 | G>T | GCA>GAA | A>E | NS | FtsX-like permease family protein |
| 96054 | NAR |  |  |  |  |
| 97936 | A>G | CTC>CCC | L>P | NS | penicillin-binding protein |
| 99892 | C>A | CCC>CAC | P>H | NS | WxL domain-containing protein |
| 102571 | C>T | TCC>TCT | S | S | YihY |
| 102721 | G>A | ATG>ATA | M>I | NS | DUF916 and DUF3324 domain-containing protein |
| 110044 | T>G>A | GCT>GCG>GCA | A | S | FAD-dependent oxidoreductase |
| 120178 | NAR |  |  |  |  |
| 128766 | G>A | GTC>GTT | V | S | ABC transporter ATP-binding protein |
| 130616 | C>T | GCA>GTA | A>V | NS | PTS glucose transporter subunit IIA |
| 131750 | T>C | ATG>ACG | M>T | NS | magnesium-translocating P-type ATPase |

SNPs: Single-Nucleotide Polymorphism; NAR: not in annotated region of genome (intergenic region); NS: non-synonymous; S: synonymous. L=Leucine; Y=Tyrosin; T=Threonine; A=Alanine; V=Valine; I=Isoleucine; K=Lysine; Q=Glutamine; N=Asparagine; P=Proline; R=Arginine; G=Glycine; S=Serine; D=Aspartic Acid; H=Histidine; E=Glutamic Acid; M=methionine. The gene product was defined according to GenBank.
